# Organizational Support in Healthcare Redesign Education: A Mixed-Methods Exploratory Study of Expert Coach and Executive Sponsor Experiences

**DOI:** 10.3390/ijerph17155308

**Published:** 2020-07-23

**Authors:** Pieter J. Van Dam, Phoebe Griffin, Gregory M. Peterson, Nicole S. Reeves, Lea Kirkwood, Sarah J. Prior

**Affiliations:** 1 Tasmanian School of Medicine, College of Health and Medicine, University of Tasmania, Hobart 7000, Tasmania, Australia; 2 Tasmanian School of Medicine, College of Health and Medicine, University of Tasmania, Launceston 7250, Tasmania, Australia; phoebe.griffin@utas.edu.au; 3 School of Pharmacy and Pharmacology, College of Health and Medicine, University of Tasmania, Hobart 7000, Tasmania, Australia; g.peterson@utas.edu.au; 4 Tasmanian School of Medicine, College of Health and Medicine, University of Tasmania, Burnie 7320, Tasmania, Australia; nicole.reeves@utas.edu.au (N.S.R.); sarah.prior@utas.edu.au (S.J.P.); 5 Agency for Clinical Innovation, New South Wales Health, St Leonards 2065, New South Wales, Australia; Lea.Kirkwood@health.nsw.gov.au

**Keywords:** project-based learning, work-integrated learning, healthcare redesign, health service improvement, organizational support, sponsor, expert coach, education, quality improvement

## Abstract

Healthcare organizations must continue to improve services to meet the rising demand and patient expectations. For this to occur, the health workforce needs to have knowledge and skills to design, implement, and evaluate service improvement interventions. Studies have shown that effective training in health service improvement and redesign combines didactic education with experiential project-based learning and on-the-ground coaching. Project-based learning requires organizational support and oversight, generally through executive sponsorship. A mixed-methods approach, comprising online surveys and semi-structured interviews, was used to explore the experiences of expert coaches and executive sponsors as key facilitators of workplace-based projects undertaken during an Australian postgraduate healthcare redesign course. Fifteen (54%) expert coaches and 37 (20%) executive sponsors completed the online survey. Ten expert coaches and six executive sponsors participated in interviews. The survey data revealed overall positive experiences for coaches and mixed experiences for sponsors. Interview participants expressed a sense of fulfillment that came from working with project teams to deliver a successful project and educational outcomes. However, concerns were raised about adequate resourcing, organizational recognition, competing priorities, and the skills required to effectively coach and sponsor. Expert coaches and executive sponsors sometimes felt under-valued and may benefit from cohort-tailored and evidence-based professional development.

## 1. Introduction

Ongoing health service improvement is required to ensure that organizations continue to provide safe, effective, and efficient patient-centered care [1]. Studies have estimated that up to 30% of the delivery of healthcare can be classified as low-value care [2], defined as “use of an intervention where evidence suggests it confers no or very little benefit on patients, or risk of harm exceeds likely benefit, or, more broadly, the added costs of the intervention do not provide proportional added benefits” [3]. For healthcare organizations to improve services, staff must have the capacity to design, implement, and analyze setting-relevant improvement interventions. Therefore, all health professionals should strive to improve their knowledge and skills in the use of healthcare redesign principles and other health service improvement methodologies [4].

Effective education and training in health service redesign and improvement combines theoretical, didactic education with experiential project-based learning and on-the-ground coaching [5,6,7]. Education and training must also align with the needs of the health system [8] and consumers, as well as local, national, and international goals for health service improvement [1]. Delivering an effective training program thus requires strong relationships between educational providers and healthcare organizations, such as hospitals, general medical practices, and community care providers. This allows for the development of a collaborative and adaptive understanding of the knowledge and skills required for better care, added value, and improved health outcomes [9,10].

Workplace projects undertaken as part of health service improvement education have the advantage of not only building relevant knowledge and skills but also delivering initiatives that benefit healthcare organizations and their patients [5]. Support and mentoring from senior leaders within healthcare organizations are essential for implementing and sustaining improvement initiatives [11]. One of the reasons why many healthcare organizations may struggle with the implementation and sustainability of change is that these organizations are considered “professional bureaucracies” [12], where individual healthcare professionals have control over the services delivered. Therefore, the coordination of improvement efforts heavily relies on peer and collegial processes [13], but more importantly, on expert coaches [14] and executive support [15,16,17].

Generally, executive support is provided in the form of the executive sponsorship of improvement projects. Executive sponsors provide essential project resources and are responsible for ensuring the delivery of successful project outcomes within budget and on time [18]. Expert coaches are highly skilled and experienced quality improvement practitioners. They act as personal trainers of project redesign teams by promoting a social learning environment that specifically facilitates healthcare redesign and health service improvement within the organization [14].

Local, organization-based expert coaches and executive sponsors are critical enablers of experiential, project-based health service improvement and redesign education. Yet, most studies examining the effectiveness of health service improvement education have focused on educational outcomes rather than the facilitators and processes that drive successful academic and project outcomes [19]. Therefore, this exploratory study sought to capture the experiences of expert coaches and executive project sponsors as key facilitators of workplace-based projects undertaken as part of a postgraduate healthcare redesign course delivered in partnership between the University of Tasmania (UTAS), Tasmania, Australia, and the Agency for Clinical Innovation (ACI) at New South Wales Health, New South Wales (NSW), Australia.

## 2. Materials and Methods

### 2.1. Setting

The postgraduate healthcare redesign course was developed as a partnership initiative between UTAS and ACI at NSW Health to build capacity for health service improvement across the public health system in NSW, Australia. The graduate certificate course commenced in February 2016 with an annual intake of approximately 80 students with varying professional backgrounds [20]. The course design enables students to learn by delivering real-world benefits through a blend of project-based, work-integrated learning and traditional didactic education. Students usually attend in project teams from their local health services. Projects encompass all aspects of health service delivery and are designed to align with organizational strategic priorities and address the Institute for Healthcare Improvement’s (IHI) Triple Aim [20].

All projects undertaken within the course are supported by local, organization-based workplace coaching from NSW Health redesign leads (expert coaches) and executive project sponsors. Didactic education is provided by knowledge experts (program facilitators) at ACI and an academic teaching team at UTAS [21]. The expert coach roles are senior service manager roles that are filled by staff who are often also graduates of this or similar courses. ACI offers coaches the opportunity to attend education and professional networking sessions delivered six times per year for one to two days. They are involved with student team selection and project identification, and lodge the project application forms. Their roles are critical to the translation of knowledge to practice in their organization. Executive sponsors are senior leaders within the students’ organizations who have managerial oversight of the areas in which the projects are conducted. Executive sponsors receive a full day briefing at the beginning of the course and at regular intervals during the program. They can access an online eLearning module on sponsorship and receive support and coaching from ACI program facilitators and expert coaches.

### 2.2. Study Design

**Figure 1:**
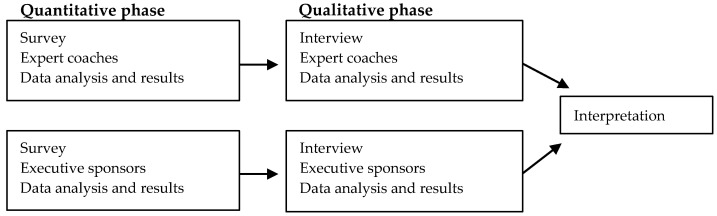
Sequential exploratory mixed-methods study design.

This study aimed to capture the experiences of expert coaches and executive project sponsors within a work-integrated postgraduate healthcare redesign course. The study employed a sequential exploratory mixed-methods research design to meet these aims (Figure 1). First, quantitative data were collected using two online surveys, one for each participant group to capture the overall expert coach and sponsor experience. As the populations of both participant groups were relatively small, qualitative data were then collected through interviews to allow for a more in-depth exploration of participant experiences. Ethical approval was obtained from the Tasmanian Health and Human Research Ethics Committee (H0017402).

### 2.3. Participants

Two groups of participants were invited to take part in the research (Table 1).

### 2.4. Study Protocol

#### 2.4.1. Surveys

The email addresses of eligible participants were obtained from the ACI (NSW) database. Each potential participant was emailed an invitation to take part in an anonymous online survey, delivered through SurveyMonkey^®^. Each participant was invited to read the information sheet at the beginning of the survey and consent was assumed to have been given when submitting the survey. The survey was open for eight weeks between 1 September 2018 and 31 October 2018.

A 20-item survey was utilized to gain demographic data and information about the experiences of expert coaches within the redesign program, and the relationship between these experiences and outcomes. A 16-item survey was utilized to obtain similar data from executive sponsors.

Three groups of questions were utilized: (i) direct questions requiring participants to select one option per question, (ii) questions requiring participants to indicate their level of agreement or disagreement with statements using a five-point Likert scale (“strongly agree” (1) to “strongly disagree” (5)), and (iii) open-ended questions inviting free-text comments.

#### 2.4.2. Interviews

Email addresses of eligible participants were obtained from the ACI (NSW) database. Each potential participant was emailed an invitation, including a participant information sheet and a consent form, to take part in a semi-structured interview to explore their experiences with the redesign program. Interview questions were informed by the research aims and the survey data. Analysis of the quantitative and qualitative (free-text comments) survey data suggested that several aspects of coach and sponsor experience warranted further exploration via semi-structured interviews. The interview questions were designed to provide a deeper understanding of the coach and sponsor experiences. For example, coaches indicated an overall high level of satisfaction with coaching project teams, yet some raised concerns regarding resourcing and professional development. Interview questions such as “How would you like to be supported as an expert coach?” and “How do you feel that the organization’s support affects student learning?” were designed to examine how the aspects in question affected the coach experience, and how they may be addressed to improve both the coach and student experiences. Participants were asked to contact the research team to express their interest in being interviewed. Interviews were conducted according to opportunity: face-to-face (expert coaches) or via telephone (executive sponsors) by a member of the research team who did not teach into the program. Interviews were conducted between January and April 2019.

### 2.5. Analyses

Quantitative survey data were analyzed using Microsoft^®^ (Microsoft Corporation, Redmond, WA, United States of America) Excel^®^. Descriptive and frequency statistics were explored.

Qualitative data were analyzed using a general inductive approach allowing a valid analysis of the raw data into summaries [23]. Data were analyzed through the process of coding, developing categories, and identifying sub-themes and major themes. Interview data were professionally transcribed and then independently coded by two members of the research team. Categories were agreed upon by both researchers and themes were identified. Quotes were sourced from raw data as supporting evidence of each major theme.

## 3. Results

### 3.1. Quantitative Data—Expert Coach Online Survey

The survey was sent to 28 redesign expert coaches and 15 (54%) completed the online survey; all (100%) respondents were female.

#### 3.1.1. Overall Experience

The survey data showed that overall, the experience of the expert coach was positive with, on average, 95% of participants responding that they agreed with the statements about their participation as a coach throughout the program (Table 2). However, some participants raised concerns around resourcing and professional development in the free-text comments:

1. My ability as a coach has been enhanced by other development opportunities I’ve undertaken not necessarily as a result of the Redesign program. As expert coaches we are not ‘taught’ how to coach, and I know the standard and style of ‘coaching/mentoring’ differs greatly between one coach and another. There is also no oversight from the program as to the efficacy of coaching.
2. In a rural LHD (Local Health District) face-to-face contact is more limited and this is affecting the type of support given.
3. It would be great to have a toolbox to use for mentoring and coaching teams.

#### 3.1.2. Organizational Support

The expert coaches indicated agreement, on average (76%), across the items related to organizational support, including their views on the role of executive sponsors (Table 3). However, mixed responses were received for the following items: sponsors were well prepared, with 21.4% disagreeing; and team members (students) were well selected, with 26.7% being neutral and 6.7% disagreeing. There was also variation in the responses to the item asking whether teams were provided with sufficient quarantined time to conduct their projects, with 26.7% being neutral and 26.7% disagreeing.

These findings were also reflected in the free-text comments:

1. Sponsorship is a significant issue and ensuring teams have enough time to get outcomes is a constant battle. Aside from detailing up-front the time commitment from sponsors, and making the sponsorship capability development ‘more mandatory’…
2. Redesign was well recognized by my Director; however, when the Director left the organization executive sponsorship was lost as the new Director supported [other]… initiatives.
3. We have consistently told teams and their managers of the time commitment required and the need for backfill, but it is never forthcoming.
4. Some sponsors are better prepared than others. Usually the sponsor gets better with repeat sponsor roles with a care focus. Ensuring team participants are released is still a struggle, as our sponsors are after faster, better, cheaper and don’t realize the effort required to make change stick.

### 3.2. Qualitative Data—Expert Coach Interviews

Ten expert coaches (36%) each participated in a face-to-face, semi-structured interview to explore their experiences of coaching students as part of the redesign course. The length of time as a redesign expert coach ranged from 12 months to 6 years, and each expert coach was from a different health district or health discipline within NSW Health.

Four major themes emerged from the interviews with the expert coaches, with several sub-themes supporting the overall findings (Table 4). These themes highlight the importance of organizational support and preparation, appropriate program structure, and clear expectations in the experience of expert coaches working with redesign students and student teams.

#### 3.2.1. Theme 1: Structured Systems Facilitated Successful Outcomes

In the experience of expert coaches, structure encompassed a wide range of factors, including the overall course structure, the project team structure, and the structured expectations around the role of the expert coaches.

Participants suggested that clear and consistent course goals lead to a more positive coaching experience as students are more likely to articulate their redesign project goals and personal academic goals in relation to their needs and expectations of the expert coach. Similarly, a further enabler for a positive coaching experience was the structured selection of appropriate workgroups and team members for each project. This included structured roles and responsibilities developed before and throughout the course and the life of the project. Coaching was therefore considered “successful” when coaching expectations were met through project teams delivering outcomes aligned with organizational strategy.

#### 3.2.2. Theme 2: Focused Interaction Built Capacity

This theme pertained to discussions around the ongoing communication between coaches and students, executive sponsors and coaches, and coaches and any external parties who had a role in the redesign project work. Participants expressed that having opportunities to build networks and form relationships within and between redesign projects led to more satisfaction in the role of being an expert coach, as well as less pressure and anxiety from an organizational perspective. Participants felt that these focused discussions helped to build capacity in clinical redesign through an open learning culture and knowledge transfer.

#### 3.2.3. Theme 3: Coaching Required Managing Competing Priorities

Participants generally described the time commitment of their coaching role as a challenge, which sometimes led to an experience that was discouraging and left them feeling unenthusiastic. However, in contrast, the need to adjust scheduling, complete marking, and utilize different methods of communication was also seen to be rewarding at the final milestones. This was demonstrated by the sense of achievement that arose from effectively managing the competing responsibilities of meeting sponsor and organizational expectations, as well as developing the teams’ skills to ensure both project and academic success. Therefore, the coaching role compelled participants to skillfully manage their capacity by balancing the project and team commitments and their own professional priorities.

#### 3.2.4. Theme 4: Team Dynamics Influenced the Coaching Experiences

The relationships within each project team were a source of pressure and frustration for some expert coaches, while others found these relationships to be a strength. Team dynamics, defined as how each team member’s role and behavior impacts and influences other team members and the team as a whole, differed from project to project. Although expert coaches had different ways of mentoring students, each indicated that they tried to work with each team in a way that met the team’s expectations and needs. However, these did not always align, and therefore, their experiences of coaching differed, depending on the dynamic within the team. Some coaches felt as though they were an integral part of their team, whilst others felt that they were more on the outside and only part of their team when required.

### 3.3. Quantitative Data—Executive Sponsor Online Survey

The survey was sent to 189 executive sponsors and 37 (20%) responses were received, with 70% being female.

#### 3.3.1. Overall Experience

The overall experience of the executive sponsors was mixed, with respondents agreeing to half of the statements about their experiences (Table 5). Executive sponsors indicated that they generally felt supported (58%), the projects were suitable (81%), projects aligned with organizational goals (70%), and the project team members appreciated the support (70%). However, less than half of sponsors felt that they were supported in their role by their organization (36%), had adequate resources available for effective sponsorship (28%), were recognized for their role (28%), and their expectations of being a sponsor were met (42%).

#### 3.3.2. Organizational Support

The executive sponsors indicated agreement, on average (86%), across the items related to organizational support, including their views on the role of expert coaches (redesign leads) (Table 6). However, some participants indicated lower levels of agreement for items related to the quarantined time for teams to conduct their projects (18% neutral to slightly disagree) and finding enough time to effectively sponsor teams (23% neutral to strongly disagree).

These findings were also reflected in the free-text comments:

1. It is probably the most worthwhile and useful program I’ve been involved in producing great change and sustainable change using great methodology and structure.
2. Reduction in funding of departmental staff meant that the original program plans could not be fully implemented.
3. The time commitment and requirements were intense.
4. The challenge is back-fill. We freed up our staff which created a significant additional cost to the organization.
5. Dysfunctional executive team… which undermined and did not support the service, therefore did not support the project team.

### 3.4. Qualitative Data—Executive Sponsor Interviews

Six executive sponsors (3%) participated in semi-structured, telephone interviews to explore their experience as a sponsor within the redesign course. Each executive sponsor was responsible for one or more projects in a different health district or health discipline across NSW between 2013 and 2017. Four major themes were identified from the interview data, with sub-themes shown in Table 7.

#### 3.4.1. Theme 1: Executive Sponsorship Was an Essential Role

Executive sponsors felt that their role was essential in several ways. The engagement with and feedback from their teams were generally positive and made them feel like a valued member of the project team, although their role was generally to provide oversight and accountability. Sponsors suggested that a positive experience was more likely to lead to valuable organizational outcomes, with improved communication, better relationships, and more commitment from those involved in the project. The amount of work required by sponsors, particularly around reviewing and engagement, was considered undesirable but necessary. Similarly, as a result of the contribution by the sponsors and the team, it was suggested that there was often disappointment at the lack of ongoing support to sustain project outcomes. This was a factor in the decision regarding whether to sponsor other teams.

#### 3.4.2. Theme 2: Organizational Evolution Required Commitment from the Entire Team

Following on from the first theme, there was significant discussion around the fact that a positive experience often resulted from good team cohesion and ongoing team collaboration. Having clearly identified responsibilities was one way in which teams were able to ensure projects moved forward, were aligned to organizational values, and resulted in motivation and positive engagement, rather than feeling like it was just about completing an academic course.

#### 3.4.3. Theme 3: Sponsors Became Emotionally Invested in the Projects and Teams

Executive sponsors shared that, at times, their role was difficult as they could see that their team was under a lot of stress, particularly regarding timing and balancing work/life/study. This contributed to the overall experience of sponsors, as their role was more about oversight and they could not step in to reduce workload or assist with completing tasks. Similarly, sponsors working with teams in rural and remote areas were impacted by the travel required by their students. There was discussion regarding how difficult it was for rural and remote students to access funding for travel and backfill for positions, and physically getting to and from airports and the emotional effects it had on the whole team. Feelings of frustration and defeat were also evident in circumstances where project sustainability was negatively affected by organizational constraints, which was something that some executive sponsors had become accustomed to but were not expecting.

#### 3.4.4. Theme 4: Sponsors Developed an Understanding of Topics

In some cases, executive sponsors expressed their satisfaction regarding improving their own knowledge through sponsoring redesign projects. This related specifically to project content and different areas of health-related expertise. This learning component often contributed to a more positive experience for sponsors as they finished with a better understanding and appreciation for the complexities within different health areas.

## 4. Discussion

The NSW health system has significantly invested in healthcare redesign capacity building by incorporating the essential roles of expert coaches and executive sponsors within the project components of the redesign course. This is in contrast to many healthcare organizations that do not possess a basic infrastructure to support quality improvement [24]. It was a positive finding that, as key stakeholders, this group’s overall levels of satisfaction with the course were high, and more importantly, that most agreed that the course had made a valuable contribution to their organization. This finding complements a consensus that the course is regarded as an important and effective capacity-building strategy by students, as previously reported [21].

Godfrey et al. [25] reported on the outcomes of an interprofessional healthcare improvement study, concluding that coaching positively supports the improvement process. However, within our study, it was found that both expert coaches and executive sponsors indicated scope for improvement in terms of organizational support and recognition of their roles, particularly in the domains of professional development and resourcing, including time allocation.

The key issue of having adequate organizational support and senior executive engagement is well-documented in the literature as a perennial barrier to successfully implementing health system improvements. For instance, when examining the introduction of electronic medical records, Haugen and Woodside [26] concluded that a lack of engagement from those chosen to lead the overall effort was the principal common factor across every organization struggling with implementation and adoption of the technology.

Previously, the experiences of expert quality improvement coaches and executive sponsors have received little attention [24], despite evidence that coaching and sponsorship contribute to effective interventions and thereby improve health services, leading to better care [25,27]. Little is known about the experience of these important professionals and how their roles are enacted within healthcare organizations. Therefore, this study has contributed to a better understanding of how quality improvement programs can be supported in the workplace by utilizing expert coaches and executive sponsors to provide the best advantage.

### 4.1. Professional Development

Both executive sponsors and expert coaches reported the need for professional development. It was mentioned by the expert coaches that the standard and style of “coaching/mentoring” differ greatly between coaches. By linking coaching practice with existing best practice and research findings, an evidence-based coaching model could contribute to improved coaching quality and credibility, leading to a better overall healthcare redesign experience [25,28]. Moreover, developing an evidence-based program of coaching to enable experienced coaches to support novice coaches could generate organizational benefits [24] regarding building capacity. Such a program would support the finding that success is dependent on the program’s structure but it requires flexibility and responsiveness to handle issues through the life-time of the project [29].

The issues experienced with redesign projects are often social in nature, as indicated by the views of the expert coaches stating that dynamics in the team played a significant role in the coaching experience. The selection of team members, creating expectations through team agreements, and liaising are vital leadership skills that expert coaches need to possess to be successful [30]. Moreover, it is important to recognize that the value of each team member’s contribution to the organization can only be realized by unlocking the full potential of that person. Therefore, the inclusion and authentic engagement of expert coaches and team members in redesign activities are essential [31,32]. This notion supports findings regarding creating opportunities to build networks and form relationships within and between redesign projects, leading to more satisfaction in the role of the expert coach. Thus, the relational aspects inherent to healthcare redesign education require attention, as observed in successful organizations who see quality improvement “not as a method, technique, discipline or skill, but as a human and organizational accomplishment” [33].

A major finding was a difference in opinion regarding preparedness for the coaching and sponsor roles. The executive sponsors agreed that the expert coaches mentored students well. However, there was no consensus that the sponsors were well prepared for their role, as expressed by the expert coaches. The performance of the executive sponsor is critical to the healthcare redesign project’s success; however, it is a role that is often assigned to a senior member of the organization who may have little knowledge or education in redesign practices [34]. The expert coaches reported that sponsors sometimes directed the team rather than listened to the team. This creates challenges for all stakeholders involved, including students, sponsors, and coaches. Many sponsors did not feel that the course supported them in their development as an executive sponsor, and this might have contributed to the overall issue of preparedness. Therefore, executive sponsors may also benefit from a comprehensive executive sponsorship program [35].

### 4.2. Organizational Support and Investment

Expanding upon the comments above relating to adequate organizational support and senior executive engagement, the lack of time and resources available to complete a redesign project were often identified by both expert coaches and executive sponsors as issues, indicating that it was not always possible to mobilize the resources needed to support students in the best possible manner. When students are nominated by their organization to undertake the course to commence a redesign project, the quarantined time to study and to carry out the project is agreed upon. However, at times, the redesign project takes place on top of their regular allocated duties and students might endure additional stress and work. It was found that this created challenges for the executive sponsor as they were unable to implement changes. Both expert coaches and executive sponsors made it clear that redesign students struggle when they do not have sufficient time, resources, and support from executive management. However, in contrast and somewhat surprisingly, Hulscher et al.’s [36] review of factors associated with successful quality improvement initiatives found no significant relationship between project success and the provision of time and resources.

Not taking away from this finding, the evidence-based practice and quality improvement literature clearly explain that the availability of time to engage in quality improvement appears to be a common barrier, which is linked to day-to-day workloads and the competing time demands of concurrently held positions [29,37]. This has implications for expert coaches and executive sponsors, who have a role to play in advocating for and negotiating adequate time and resources for student projects from the organizational senior executive group. By valuing and investing in strategic health service improvement, an engaged senior executive group can help to create an environment that facilitates successful projects [38].

### 4.3. Limitations

This study has several limitations. First, as the survey was self-reported and both cohorts potentially had an invested interest in the program, we cannot fully exclude bias. Second, the participating sample of executive sponsors was relatively small, which may affect the generalizability of the findings. The small samples also limited the scope for more sophisticated statistical analyses and further data categorization that may have provided information about how the experiences of coaches and sponsors were influenced. Third, this study explored the experiences of expert coaches and executive sponsors but did not directly investigate organizational processes and characteristics that support workplace health service improvement projects. It also remains largely unknown what the return on investment is for redesign capacity building at a health system level [39]. Further studies are needed to determine the outcomes of the course in terms of improvement in the quality and efficiency of care. Additional research is also required to investigate contextual barriers and enablers of successful projects and academic outcomes.

## 5. Conclusions

Effective coaching and sponsorship require commitment and a unique set of skills and knowledge. Cohort-tailored and evidence-based training courses are required to develop expert coaches and executive sponsors by increasing their ability to negotiate the required resources and to support students in the best possible way to carry out their projects. Healthcare organizations that want to see positive change need to recognize that developing successful expert coaches and executive sponsors requires significant effort and skill development.

The course holds great promise for facilitating the successful dissemination of redesign methods throughout health services in Australia, provided that organizational support is in place. The roles of both expert coaches and executive sponsors are critical for building the capacity for health service improvement and should be recognized and nurtured.

## Figures and Tables

**Table 1:** Roles and responsibilities of the participant groups.

Group 1	Group 2
Expert Coaches	Executive Sponsors
(*n* = 28)	(*n* = 189)
As experts in the field, they provide mentoring and coaching to students (project teams), stakeholders, and executive sponsors such that the knowledge recipients can apply the redesign body of knowledge to their project work. They build and use local networks to influence project outcomes. Expert coaches may also teach face-to-face parts of the program, provide formative feedback on project deliverables, and contribute to course reviews and enhancements.	These are persons with high levels of influence within their organizations who are accountable for the project, securing any required resources, and helping to remove organizational barriers [22]. They govern the project direction and decisions, keep the project aligned with strategic aims, manage executive stakeholder relationships through mechanisms, such as steering committees, and manage escalated project risks and issues.

**Table 2:** Experiences of expert coaches (*N* = 15).

Statements	Strongly Agree	Slightly Agree	Neutral	Slightly Disagree	Strongly Disagree
The program has helped improve my own capacity to mentor others	8 (53%)	4 (27%)	2 (13%)	1 (7%)	0 (0%)
I felt supported as a coach and mentor by the program facilitators	8 (53%)	7 (47%)	0 (0%)	0 (0%)	0 (0%)
I felt supported as a coach by my organization	6 (40%)	7 (47%)	2 (13%)	0 (0%)	0 (0%)
I had adequate resources available to effectively mentor team members	7 (47%)	6 (40%)	2 (13%)	0 (0%)	0 (0%)
I felt the projects were well suited to the redesign methodology	7 (47%)	8 (53%)	0 (0%)	0 (0%)	0 (0%)
I felt that team members appreciated my mentorship	13 (87%)	2 (13%)	0 (0%)	0 (0%)	0 (0%)

**Table 3:** Perceptions of expert coaches regarding organizational support (*N* = 15).

Statements	Strongly Agree	Slightly Agree	Neutral	Slightly Disagree	Strongly Disagree
The project/s was well aligned with local health service priorities	9 (60%)	6 (40%)	0 (0%)	0 (0%)	0 (0%)
Sponsors were well prepared for their role *	4 (29%)	5 (36%)	2 (14%)	2 (14%)	1 (7%)
I felt that team members were well selected	4 (27%)	6 (40%)	4 (27%)	1 (7%)	0 (0%)
I was able to negotiate the resources my team/s required for their project/s	3 (20%)	9 (60%)	1 (7%)	2 (13%)	0 (0%)
The team/s were provided with sufficient quarantined time to conduct their project/s	3 (20%)	4 (27%)	4 (27%)	4 (27%)	0 (0%)
I was provided with enough time to mentor and guide project teams	6 (40%)	7 (47%)	1 (7%)	1 (7%)	0 (0%)
My organization values the redesign program	8 (53%)	5 (33%)	1 (7%)	1 (7%)	0 (0%)
My organization recognized my team/s achievements	6 (40%)	5 (33%)	3 (20%)	1 (7%)	0 (0%)

* Note: There were only 14 respondents to this question.

**Table 4:** Themes from expert coach interviews.

Major Theme	Sub-Theme(s)	Quotes
Coaching success is dependent on the structure	Course structure plays a major role in the way students learn from their mentors A blended learning model approach is beneficial for delivering redesign project initiatives Pre-selection of appropriate workgroups is essential for success Information provision is a key component of managing a course The role of a mentor must be clear	“Setting the stage before each project is important” “My role is around making sure people have skills in project management and improvement methodologies” “Trying to articulate where I fit in is a challenge”
Focused interaction builds capacity	Building collegial networks plays a role in educational satisfaction The success of redesign projects is influenced by the sum of all parts Fostering an open learning culture provides a good opportunity for skills development and knowledge transfer Working as part of a multi-level, multi-disciplinary team creates a professional and educational support network	“Negotiating with managers on an ongoing basis can be challenging” “I’ve got my network outside of here which is really valuable” “It’s more than mentoring… it’s about getting your hand dirty, actually get in and do it with them” “People learn more from each other”
Coaching demands a shift in priorities	Competitive and demanding professional roles can be a barrier to learning opportunities and positive outcomes for students and mentors Both workplace and academic support are requirements for redesign project completion and success Coaching is a big commitment Scheduling can determine outcomes	“A challenge as coaches is just keeping up” “Some sponsors want what they want…it affects the morale of the team” “Capacity waxes and wanes”
Team dynamics influence coaching experiences	A solid knowledge base is a priority for providing valuable feedback Personal contact and communication are necessary tools for engaging with students Learning about teams means learning about needs and expectations A successful approach to coaching is individualized and driven by needs	“Different skill sets and different levels of knowledge…frustration comes out” “I feel really valuable” “I don’t like to tell people what to do…I like a hovering approach”

**Table 5:** Experiences of executive sponsors (*N* = 37).

Statements	Yes Responses *n* (%)
I felt supported as a sponsor by program facilitators	21 (58%)
I felt supported as a sponsor by my organization	13 (36%)
I had adequate resources available to effectively sponsor team members	10 (28%)
I felt the projects were suitable for the program	29 (81%)
I felt that projects generally aligned with organizational goals	25 (69%)
I felt that team members appreciated my sponsorship	25 (69%)
My work as a team sponsor was recognized	10 (28%)
My expectations of sponsoring project teams were met	15 (42%)

**Table 6:** Perceptions of executive sponsors regarding organizational support (*N* = 37).

Statements	Strongly Agree	Slightly Agree	Neutral	Slightly Disagree	Strongly Disagree
The project/s was well aligned with local health service priorities	29 (78%)	6 (16%)	2 (5%)	0 (0%)	0 (0%)
Redesign leads (expert coaches) mentored the project teams well	29 (78%)	6 (16%)	2 (5%)	0 (0%)	0 (0%)
The right team members were well selected	24 (65%)	8 (22%)	4 (11%)	0 (0%)	1 (3%)
I was able to negotiate the resources my team/s required for their project/s	15 (41%)	15 (41%)	3 (8%)	3 (8%)	1 (3%)
The team/s were provided with sufficient quarantined time to conduct their project/s	18 (49%)	12 (32%)	2 (5%)	5 (13%)	0 (0%)
I could find enough time to effectively sponsor project teams	8 (22%)	20 (54%)	2 (5%)	5 (13%)	2 (5%)
My organization values the redesign program	22 (59%)	9 (24%)	5 (13%)	1 (3%)	0 (0%)
My organization recognized my team/s’ achievements	19 (51%)	14 (38%)	2 (5%)	1 (3%)	1 (3%)

**Table 7:** Themes from executive sponsor interviews.

Major Theme	Sub-Theme(s)	Quotes
Executive sponsorship is an essential role	Sponsors provide oversight and accountability A workplace sponsor can build and develop relationships Good organizational support leads to good organizational alignment Sponsor engagement is important for project planning, identifying issues, devising solutions, and implementation Project development and successful completion benefits from organizational commitment Sponsor preparation should be a priority Sponsor value is determined by knowledge and engagement	“The role of a sponsor should be crystal clear” “The volume of work was daunting—Only negative” “It gives liberty to the project and …students enjoy having that additional contact where they probably wouldn’t have that in their current role” “You (sponsors) can never have enough training and support”
Organizational evolution requires commitment from the entire team	Work-integrated learning is beneficial for achieving organizational goals Sponsors facilitate the transfer of learning and skills through executive relationships Formal outcome measurement provides a holistic interpretation of projects Innovation is evident in executive sponsor commitment to work-integrated learning	“Sponsors are for risk management” “Teams get a sponsor… It’s a positive way to make change” “We’re still reaping the benefits”
Sponsors become emotionally invested in projects and teams	The pressure to deliver outcomes influences experience Good foundations create highly skilled and competent change leaders Knowing the audience leads to better learning outcomes Relevant and appropriate resources can improve what people take away from a course Project sustainability can be an issue when external support is withdrawn	“I had to be aware of the workload of the students and the stress that they were under” “It’s a massive amount of travel for students…up to 8 h just to get to an airport to attend face-to-face days” “Unless someone is monitoring it all the time…it loses a lot of its impact” “It’s frustrating and energy sapping…you’ve seen it all before”
Projects provide sponsors with opportunities to develop specific content knowledge	Sponsors ensure that project priorities align with organizational views and strategies Sponsor input is a strength Workplace relevance is vital for ongoing project success Completing a work-integrated learning project creates a sense of satisfaction and accomplishment	“It was very beneficial for me, to get a handle of the health and complex care needs of the community” “The beauty of what we did…what we can do with existing software” “It was very educational for me at the beginning…the whole integrated care space”
